# First-line monodrug chemotherapy in low-risk gestational trophoblastic neoplasia: a network meta-analysis

**DOI:** 10.3389/fonc.2023.1276771

**Published:** 2024-01-05

**Authors:** Fang Zhou, Li Kemin

**Affiliations:** ^1^ West China University Hospital of Sichuan University, Chengdu, Sichuan, China; ^2^ The Department of Obstetrics and Gynecology, West China Second University Hospital of Sichuan University, Chengdu, Sichuan, China; ^3^ Key Laboratory of Birth Defects and Related Diseases of Women and Children (Sichuan University), Ministry of Education, Chengdu, Sichuan, China

**Keywords:** first-line chemotherapy, low-risk gestational trophoblastic neoplasia, monodrug, network meta-analysis, GTN

## Abstract

**Objective:**

The efficacy of the first-line monodrug chemotherapy has been generally established for low-risk GTN. Most patients can achieve a complete response after the first-line monodrug chemotherapy. However, which monodrug chemotherapy regimen is better for individual patients with GTN is not yet certain. This study aimed to assess the efficacy of first-line monodrug chemotherapy in low-risk gestational trophoblastic neoplasia (GTN).

**Method:**

Databases, including PubMed, Embase, Web of Science, and Cochrane Library, were searched from inception to November 1, 2022, for case–control studies on first-line monodrug chemotherapy in GTN. Network meta-analysis was performed to compare the efficacy outcome of six monodrug chemotherapy regimens in GTN, with a complete response rate as the endpoint.

**Result:**

Twenty-four studies were considered eligible, including 9 randomized controlled trials (RCTs) and 15 non-RCTs. A total of 3344 patients with low-risk GTN were involved. Six monodrug chemotherapy regimens were included and analyzed. In descending order of efficacy, these six regimens were VP-16 (5 days), ACT-D (5 days), MTX (5 days), ACT-D (1.25 mg/m^2^), MTX (8 days), and MTX (30–50 mg/m^2^) in all study, and five regimens were ACT-D (5 days), MTX (5 days), ACT-D (1.25 mg/m^2^), MTX (8 days), and MTX (30–50 mg/m^2^) in RCT.

**Conclusion:**

Among the six first-line monodrug chemotherapy regimens for low-risk GTN in all study, VP-16 (5 days) was the best in terms of efficacy. And five regimens in RCT, ACT-D was the best. However, the finding needs to be validated through more high-quality clinical studies.

## 1 Introduction

Gestational trophoblastic neoplasia (GTN) is a rare malignancy originating from placental trophoblasts. Despite the high metastatic potential and lethal risk, GTN is associated with a response rate as high as 90% under most situations (1, 2). The International Federation of Gynecology and Obstetrics (FIGO)/World Health Organization (WHO) prognosis scoring system (2000) classifies GTN into low risk (≤6 points), high risk (>6–12 points), and ultra high risk (≥13 points), for which stratified treatment is recommended.

The FIGO 2021 guidelines (3) recommend monodrug chemotherapy for low-risk GTN and combination chemotherapy for high-risk GTN. For the former, the commonly used first-line agents are methotrexate (MTX) and actinomycin D (ACT-D). However, which monodrug or chemotherapy regimen is the best for individual patients has not yet been established. An intramuscular injection of MTX is a convenient and widely used MTX dosing regimen due to the prevalence of day care wards and family doctors in foreign countries. In China, textbooks recommend the 5-fluorouracil (5-FU)/floxuridine regimen. Even today, some Chinese grassroots-level hospitals, or even grade-3 first-class hospitals, are still using this regimen, although it has already been removed from the 2015 FIGO guidelines (4). The reason is that 5-FU is usually given for a long period, causing obvious adverse and toxic effects, for example, severe bone marrow suppression and ulceration of the intestinal mucosa, further leading to diarrhea. In some serious cases, pseudomembranous colitis induced by *Staphylococcus aureus* may even occur, leading to death. Seven monodrug chemotherapy regimens are more commonly used in low-risk GTN: (1) MTX (8 days) regimen: MTX 1 mg/kg or 50 mg, intramuscular (IM) or intravenous (IV), on days 1, 3, 5, and 7; FA 0.1 mg/kg, IM or oral, on days 2, 4, 6, and 8; (2) MTX (5 days) regimen: MTX 0.4 mg/kg or 25 mg, IM or IV, for 5 days consecutively; (3) ACT-D (1.25 mg/m^2^) regimen: ACT-D 1.25 mg/m^2^, intravenous injection (ivgtt;2 mg at most); (4) ACT-D (5 days) regimen: ACT-D 10–12 μg/kg or 0.5 mg, ivgtt, for 5 days consecutively; (5) MTX (30–50 mg/m^2^) regimen: MTX 30–50 mg/m^2^, IV; (6)VP-16 (5 days) regimen:VP-16 100 mg/m^2^.d;(7) MTX pulse regimen: MTX 100 mg/m^2^ IV, then 200 mg/m^2^ ivgtt (over 12h); FA 15 mg. Six monodrug chemotherapy regimens are shown in **Table 1**.

**Table 1:** First-line monodrug chemotherapy regimens.

ACT-D (5 days)	10–12 μg/kg or 0.5 mg, ivgtt, for 5 days consecutively
MTX (8 days)	1 mg/kg or 50 mg, IM or IV, on days 1, 3, 5, and 7; FA 0.1 mg/kg, IM or oral, on days 2, 4, 6, and 8
ACT-D (1.25 mg/m2)	1.25 mg/m^2^, intravenous injection (ivgtt;2 mg at most)
MTX (5 days)	0.4 mg/kg or 25 mg, IM or IV, for 5 days consecutively
VP-16 (5 days)	VP-16 100 mg/m^2^.d, for 5 days consecutively
MTX (30–50 mg/m2)	30–50 mg/m^2^
MTX pulse regimen	100 mg/m^2^ IV, then 200 mg/m^2^ ivgtt (over 12h); FA 15 mg

The efficacy of the first-line monodrug chemotherapy has been generally established for low-risk GTN. Most patients can achieve a complete response after the first-line monodrug chemotherapy. However, which monodrug chemotherapy regimen is better for individual patients with GTN is not yet certain. A meta-analysis (5) comparing several first-line chemotherapy regimens included 7 RCTs, involving 667 patients with low-risk GTN. The results showed that ACT-D was significantly better than MTX in terms of effectiveness. The pulsed chemotherapy regimens using ACT-D and MTX did not differ significantly in side effects. Nevertheless, more high-quality evidence is needed to treat low-risk GTN. Given the diversity of the treatment regimens, a network meta-analysis can inform the choice of the optimal regimen for this condition. We performed a network meta-analysis with a complete response rate after the first-line monodrug chemotherapy to offer clues for the clinical choice of the chemotherapy regimen.

## 2 Data and method

Network meta-analysis was performed according to the PRISMA guidelines (6, 7).

### 2.1 Literature retrieval

**Figure 1:**
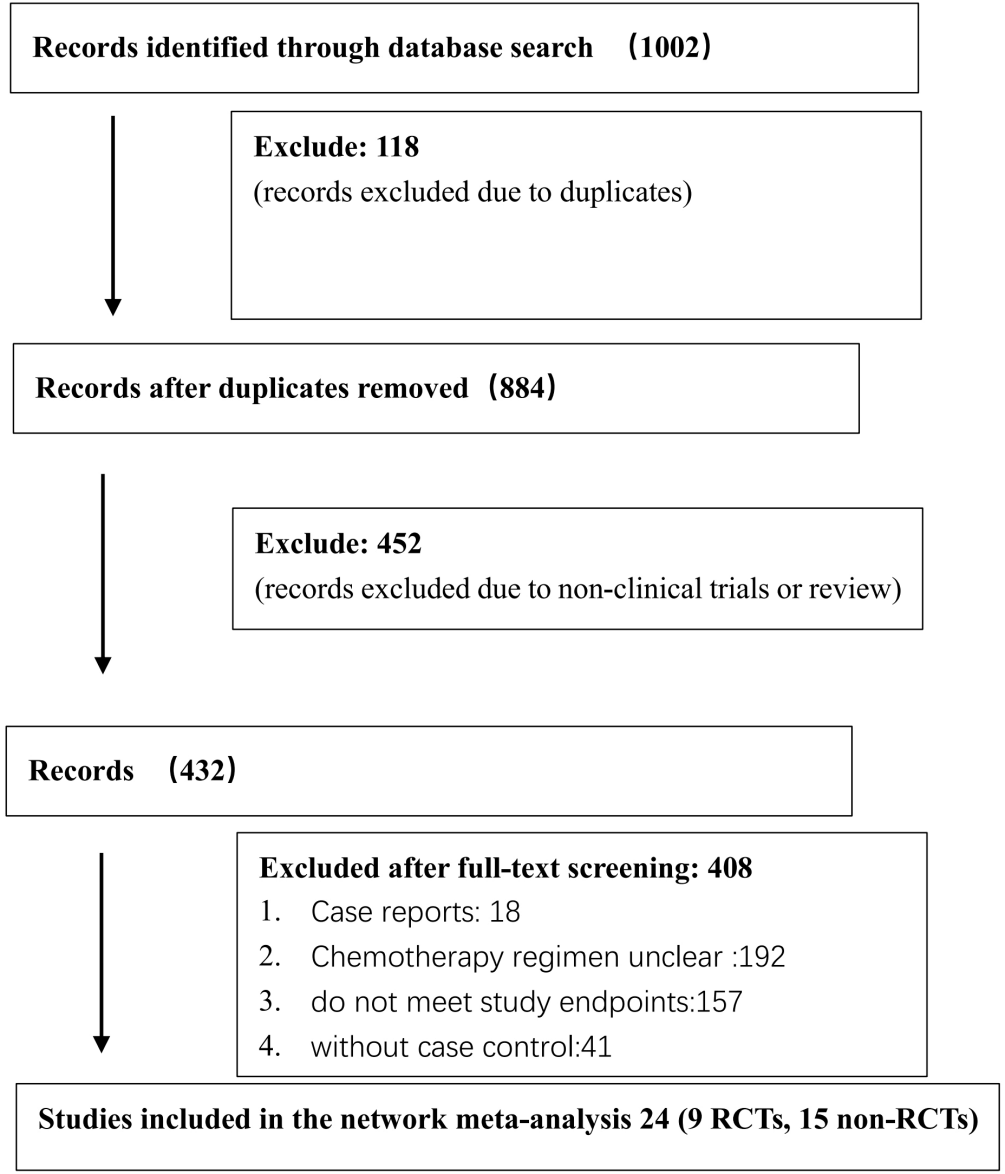
The flow diagram of patients.

The Cochrane Library, PubMed, Embase, and Web of Science databases were searched using the Medical Subject Heading (MeSH)-term search strategy from inception to November 1, 2022. Literature search and screening were conducted by two researchers independently. The divergence of opinions was settled through a discussion between the two researchers. If the problem still persisted, a third researcher specialized in methodology was invited. The search words included the following: low-risk or low risk, gestational trophoblastic neoplasia, or gestational trophoblastic tumor. The flowchart of the literature search and screening is shown in **Figure 1**.

### 2.2 Inclusion and exclusion criteria

The inclusion and exclusion criteria were developed based on the Population, Intervention, Comparison, Outcomes, and Studies principles. The participants were confirmed with low-risk GTN, with a prognostic score ≤6 according to the FIGO/WHO prognostic scoring system; the studies were RCTs or non-RCTs; and the interventional treatments were first-line monodrug chemotherapy regimens, which included but were not confined to the following: MTX (8 days) regimen, MTX (5 days) regimen, ACT-D (1.25 mg/m^2^) regimen, ACT-D (5 days) regimen, MTX (30–50 mg/m^2^) regimen, and MTX pulsed regimen. The primary endpoint was the complete response rate after the first-line monodrug chemotherapy. Published studies written in English were included.

Studies without controls, studies containing an unclear description of the chemotherapy regimens, studies using oral chemotherapy regimens as interventional treatment, and studies from which important endpoint data could not be extracted were excluded.

### 2.3 Data extraction

Two researchers independently extracted the following data from the included studies: authors, publication time, study site, study type, interventional treatment, subjects, sample size, and age. The complete response rate after the first-line monodrug chemotherapy was the outcome measure. Randomization scheme, blinding, and reporting were used as methodological indicators. The divergence of opinions was settled through a discussion between two researchers. If the problem still persisted, a third researcher specialized in methodology was invited.

### 2.4 Bias and quality assessment of the included studies

Bias and quality assessment was conducted for RCTs using Cochrane collaboration’s tool for assessing the risk of bias (8). The following seven categories of indicator data were included for bias and quality assessment: (1) random sequence generation (selection bias); (2) allocation concealment (selection bias); (3) blinding of participants and personnel (performance bias); (4) blinding of outcome assessment (detection bias); (5) incomplete outcome data (attrition bias); (6) selective reporting (reporting bias); and (7) other bias. Bias and quality assessment was conducted for the included non-RCTs using the quality assessment tools for observational studies (9). The following 11 categories of indicator data were included for bias and quality assessment: (1) the source of information was defined (survey and record review), (2) inclusion and exclusion criteria were listed for exposed and unexposed participants (cases and controls) or previous publications were referred to; (3) time period used for identifying patients was indicated; (4) whether participants were consecutive if not population based was indicated; (5) whether evaluators of subjective components of study were masked to other aspects of the status of the participants was indicated; (6) any assessments undertaken for quality assurance purposes were described (e.g., test/retest of primary outcome measurements); (7) any patient exclusions from analysis were explained; (8) how confounding was assessed and/or controlled was described; (9) if applicable, how missing data were handled in the analysis were explained; (10) patient response rates and completeness of data collection were summarized; and (11) what follow-up, if any, was expected and the percentage of patients for which incomplete data or follow-up was obtained were clarified. Bias and quality assessment was conducted for each study based on the aforementioned criteria.

### 2.5 Data analysis

The data on the sample size and complete response rate under different interventional treatments were extracted from the included studies. Then, network meta-analysis was performed using R 4.2.2. The odds ratio was calculated for the enumeration data, and the measurements were expressed as mean ± standard deviation. First, the chi-square test for homogeneity was performed. *I* ^2^ ≤50% indicated small heterogeneity, and the study was considered eligible for meta-analysis. *I* ^2^ >50% indicated large heterogeneity. Thus, the sources of heterogeneity were identified and removed before the meta-analysis. Network analysis was performed by running the Markov Chain Monte Carlo algorithm.

## 3 Results

### 3.1 Basic features of the included studies

The search strategy was developed using the MeSH terms. The preliminary screening yielded 1002 studies, among which repeated studies and those not eligible for the meta-analysis were excluded, resulting in 24 eligible ones (10–33). Specifically, 9 RCTs (10, 14, 16, 17, 20, 21, 23, 24, 33) and 15 non-RCTs (11–13, 15, 18, 19, 22, 25–32) were finally included. A total of 3344 low-risk patients were involved. The following six first-line monodrug chemotherapy regimens were involved in studies: MTX (8 days) regimen, MTX (5 days) regimen, ACT-D (1.25 mg/m^2^) regimen, ACT-D (5 days) regimen, MTX(30–50 mg/m^2^) regimen, and VP-16 (5 days) regimen. The basic features of the included studies are presented in **Table 2**.

**Table 2:** Basic characteristics of research.

study	type	regions	treatment regiments							
			group1	sample	group2	sample	group3	sample	group4	sample
Lertkhachonsuk A 2009 (10)	RCT	Thailand	1	22	2	27				
Lee YJ 2017 (11)	retrospective study	Korea	2	53	3	18	1	5		
Matsui H 1998 (12)	retrospective study	Japan	4	192	5	126	1	46	2	36
Baptista AM 2012 (13)	prospective study	Brazil	2	20	1	20	1	20		
Gilani MM 2005 (14)	RCT	Iran	3	18	6	28				
Mu X 2018 (15)	retrospective study	China	3	34	1	26				
Mousavi A 2012 (16)	RCT	Iran	3	50	4	25				
Shahbazian N 2014 (17)	RCT	Iran	3	15	6	15				
Korkmaz V 2022 (18)	retrospective study	Turkey	2	53	6	20				
Maestá I 2018 (19)	retrospective study	Brazil	2	151	6	174				
Kang HL 2019 (20)	RCT	China	1	49	4	59				
Yarandi F 2016 (21)	RCT	USA	4	32	3	30				
Schorge JO 2003 (22)	prospective study	USA	2	5	4	20	6	7		
Osborne RJ 2011 (23)	RCT	Canada	6	107	3	109				
Yarandi F 2008 (24)	RCT	Iran	6	81	3	50				
Hoskins PJ 2020 (25)	retrospective study	Canada	3	100	6	97				
Abrão RA 2008 (26)	retrospective study	Brazil	1	42	4	42				
Uberti EMH 2015 (27)	retrospective study	Brazil	2	115	3	79				
Roberts JP 1996 (28)	retrospective study	USA	1	4	4	61				
Kang WD 2010 (29)	retrospective study	Korea	2	59	6	48				
Matsui H 2005 (30)	retrospective study	Japan	2	24	4	132	5	90	1	25
Fülöp V 2021 (31)	retrospective study	Hungary	2	304	1	109				
Xu J 2022 (32)	retrospective study	China	1	88	4	122				
Anfinan N.M 2020 (33)	RCT	Jeddah	2	26	6	34				

group: 1. ACT-D: 10 Kg/kg per day intravenously for 5 days, every 2 weeks, 2. MTX: 1 mg/kg per day on days 1, 3, 5, and 7, alternating with intramuscular folinic acid 0.1 mg/kg per day on days 2, 4, 6, and 8, every two weeks, 3. ACT-D: pulse actinomycin-D (1.25 mg/m2 ) once every 14 days with a maximum dose of 2 mg, 4. MTX: 0.4mg/kg 5 days, 5. VP-16: 2.0mg/kg 5 days, 6. MTX: 30 -50 mg/m2 weekly.

### 3.2 Bias and quality assessment of the included studies

The results of bias and quality assessment of the 9 RCTs are shown in **Table 3**, and those of the 15 non-RCTs are shown in **Table 4**. The included studies were mostly clinical trials of medium quality.

**Table 3:** Assessment of risk of bias (RCT).

study	1	2	3	4	5	6	7	Jadad score
Lertkhachonsuk A 2009 (10)	Y	Y	unclear	unclear	unclear	Y	Y	4
Gilani MM 2005 (14)	unclear	Y	unclear	unclear	unclear	Y	Y	3
Mousavi A 2012 (16)	unclear	Y	unclear	unclear	unclear	Y	Y	3
Shahbazian N 2014 (17)	unclear	Y	unclear	unclear	unclear	Y	Y	3
Kang HL 2019 (20)	Y	Y	unclear	unclear	unclear	Y	Y	4
Yarandi F 2016 (21)	Y	Y	Y	Y	unclear	Y	Y	6
Osborne RJ 2011 (23)	Y	Y	unclear	unclear	unclear	Y	Y	4
Yarandi F 2008 (24)	unclear	Y	unclear	unclear	unclear	Y	Y	3
Anfinan N.M 2020 (33)	Y	Y	unclear	unclear	unclear	Y	Y	4

1. random sequence generation, 2. allocation concealment, 3. blinding of participants and personnel, 4. blinding of outcome assessment, 5. incomplete outcome data, 6. selective reporting, 7. other bias.

**Table 4:** Assessment of risk of bias (non-RCT).

study	1	2	3	4	5	6	7	8	9	10	11	score
Lee YJ 2017 (11)	Y	Y	Y	N	N	N	Y	Y	Y	Y	Y	8
Matsui H 1998 (12)	Y	Y	Y	N	N	N	Y	Y	Y	Y	Y	8
Baptista AM 2012 (13)	Y	Y	Y	N	N	N	Y	Y	Y	Y	Y	8
Mu X 2018 (15)	Y	Y	Y	N	N	N	Y	Y	Y	Y	Y	8
Korkmaz V 2022 (18)	Y	Y	Y	N	N	N	Y	Y	Y	Y	Y	8
Maestá I 2018 (19)	Y	Y	Y	N	N	N	Y	Y	Y	Y	Y	8
Schorge JO 2003 (22)	Y	Y	Y	N	N	N	Y	Y	Y	Y	Y	8
Hoskins PJ 2020 (25)	Y	Y	Y	N	N	N	Y	Y	Y	Y	Y	8
Abrão RA 2008 (26)	Y	Y	Y	N	N	N	Y	Y	Y	Y	Y	8
Uberti EMH 2015 (27)	Y	Y	Y	N	N	N	Y	Y	Y	Y	Y	8
Roberts JP 1996 (28)	Y	Y	Y	N	N	N	Y	Y	Y	Y	Y	8
Kang WD 2010 (29)	Y	Y	Y	N	N	N	Y	Y	Y	Y	Y	8
Matsui H 2005 (30)	Y	Y	Y	N	N	N	Y	Y	Y	Y	Y	8
Fülöp V 2021 (31)	Y	Y	Y	N	N	N	Y	Y	Y	Y	Y	8
Xu J 2022 (32)	Y	Y	Y	N	N	N	Y	Y	Y	Y	Y	8

1. Define the source of information (survey, record review), 2. List inclusion and exclusion criteria for exposed and unexposed subjects (cases and controls) or refer to previous publications, 3. Indicate time period used for identifying patients, 4. Indicate whether or not subjects were consecutive if not population-based, 5. Indicate if evaluators of subjective components of study were masked to other aspects of the status of the participants, 6. Describe any assessments undertaken for quality assurance purposes (e.g., test/retest of primary outcome measurements), 7. Explain any patient exclusions from analysis, 8. Describe how confounding was assessed and/or controlled, 9. If applicable, explain how missing data were handled in the analysis, 10. Summarize patient response rates and completeness of data collection, 11. Clarify what follow-up, if any, was expected and the percentage of patients for which incomplete data or follow-up was obtained.

### 3.3 Complete response rate of first-line monodrug chemotherapy regimens in low-risk GTN

Six studies compared the ACT-D (5 days) regimen and the MTX (8 days) regimen; two studies compared the ACT-D (5 days) regimen and ACT-D (1.25 mg/m^2^) regimen; two studies compared the MTX (8 days) regimen and the ACT-D (1.25 mg/m^2^) regimen; five studies compared the ACT-D (5 days) regimen and the MTX (5 days) regimen; two studies compared the ACT-D (5 days) regimen and the VP-16 (5 days) regimen; two studies compared the MTX (5 days) regimen and the VP-16 (5 days) regimen; five studies compared the ACT-D (1.25 mg/m^2^) regimen and the MTX (30–50 mg/m^2^) regimen; three studies compared the ACT-D (1.25 mg/m^2^) regimen and the MTX (5 days) regimen; five studies compared the MTX (8 days) regimen and the MTX (30–50 mg/m^2^) regimen; two studies compared the MTX (8 days) regimen and the MTX (5 days) regimen; and one study compared the MTX (5 days) regimen and the MTX (30–50 mg/m^2^) regimen.

**Figure 2:**
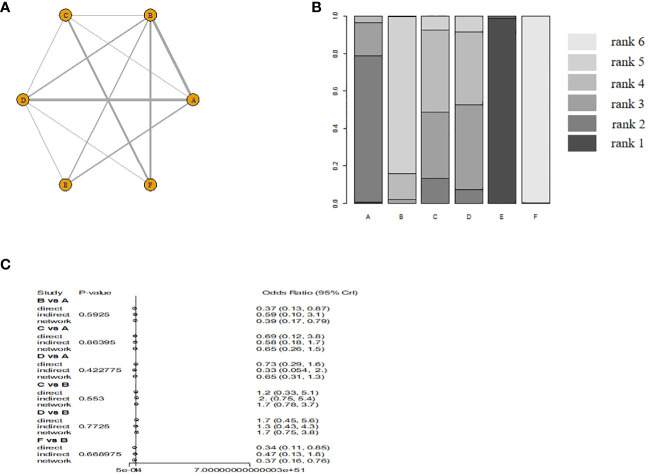
The evidence graph and the results of network meta-analysis in all study **(A)** The evidence graph, **(B)** ranks of treatments, **(C)** forest of network meta-analysis). A: ACT-D (10 ug/kg per day intravenously for 5 days,every 2 weeks), B: MTX(1 mg/kg per day on days 1, 3, 5, and 7, alternating with intramuscular folinic acid 0.1 mg/kg per day on days 2, 4, 6, and 8, every two weeks), C: pulse Act-D(pulse actinomycin-D (1.25 mg/m2) once every 14 days with a maximum dose of 2 mg), D: MTX(0.4mg/kg 5 day), E: VP-16(2.0mg/kg 5 day), F: MTX(30 mg/m2/weekly).

**Figure 3:**
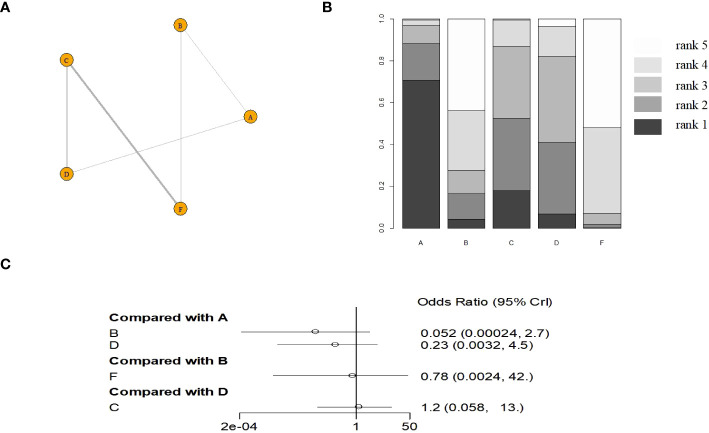
The evidence graph and the results of network meta-analysis in RCT **(A)** The evidence graph, **(B)** ranks of treatments, **(C)** forest of network meta-analysis). A: ACT-D (10 ug/kg per day intravenously for 5 days,every 2 weeks), B: MTX(1 mg/kg per day on days 1, 3, 5, and 7, alternating with intramuscular folinic acid 0.1 mg/kg per day on days 2, 4, 6, and 8, every two weeks), C: pulse Act-D(pulse actinomycin-D (1.25 mg/m2) once every 14 days with a maximum dose of 2 mg), D: MTX(0.4mg/kg 5 day), E: VP-16(2.0mg/kg 5 day), F: MTX(30 mg/m2/weekly).

Six monodrug chemotherapy regimens were included and analyzed. The probability ranking results show that VP-16 (5 days) is most likely to be the most effective treatment option. The probability is about 99%, followed by ACT-D (5 days)(78%), MTX (5 days)(45%), ACT-D (1.25 mg/m2)(43%). Subgroup analysis found ACT-D (5 days) is most likely to be the most effective treatment option, MTX (5 days), ACT-D (1.25 mg/m^2^), MTX (8 days), and MTX (30–50 mg/m^2^) in RCT(there are no RCTs on VP-16). The evidence graph and the results of network meta-analysis in all study are shown in **Figure 2**. The evidence graph and the results of network meta-analysis in RCT are shown in **Figure 3**.

Network meta-analysis has good consistency with traditional meta-analysis, the efficacy value and ranking probability do not change significantly, and the results are stable. GTN-node splitting analysis of inconsistency are presented in **Supplementary Figure S1** and **Supplementary Table S1**. The analysis of heterogeneity are shown in **Supplementary Figure S2** and **Supplementary Table S2**.

## 4 Discussion

The FIGO guidelines (3) recommend monodrug chemotherapy for low-risk GTN, and the options include MTX and ACT-D. The hCG level should be monitored once every 2 weeks before each cycle to guide the subsequent treatment. If the hCG level drops to normal after chemotherapy, two to three cycles of chemotherapy should be given before discontinuation. If a satisfactory initial response to chemotherapy is followed by a reduction in the hCG level to the plateau (down by <10% after three cycles of chemotherapy) or first a decrease and then an increase (hCG < 1000 µ/L), the regimen should be changed to the one different from the initial treatment. If MTX has been previously used, the monodrug therapy should be changed to ACT-D and vice versa.

Although MTX and ACT-D monodrug chemotherapy regimens are recommended as the preferred treatments by international guidelines, which one is better for individual patients is not yet certain. Studies have been conducted on combination therapies or other first-line monodrug chemotherapy regimens, but the controversy regarding the optimal dosing regimen for chemotherapy continues. Matsui et al. (12) compared the efficacy of MTX, VP-16, and ACT-D in 247 patients with low-risk GTN. The result showed that the complete response rate of the MTX (5 days) regimen, VP-16 (5 days) regimen, ACT-D (5 days) regimen, and MTX (8 days) regimen was 73.6%, 90.1%, 84.0%, and 60.0%, respectively. The complete response rate was significantly higher for the VP-16 and ACT-D regimens than for the other two conventional regimens. The complete response rate was significantly higher for the VP-16 regimen than for the other three regimens. Maestá et al. (19) analyzed the efficacy of different MTX dosing regimens in 325 patients with low-risk GTN, namely, MTX (30–50 mg/m^2^) and MTX (8 days) regimens. Compared with the MTX (30–50 mg/m^2^) regimen, the MTX (8 days) regimen was found to have a higher sustained response rate (84% vs 62%, *P* < 0.001). Although the latter also had a higher incidence of adverse reactions, nearly all of these reactions were controllable. The MTX (8 days) regimen was superior to the MTX (30–50 mg/m^2^) regimen. Xu et al. (32) evaluated the efficacy of the ACT-D (5 days) regimen against the MTX (5 days) regimen in low-risk GTN. The results showed that the complete response rate was 72.73% in the ACT-D (5 days) regimen and 75.41% in the MTX (5 days) regimen, indicating no significant difference. Compared with the ACT-D group, the MTX monodrug group significantly reduced in the total number of chemotherapy cycles and average hospitalization cost (*P* < 0.05). No serious adverse reactions were reported in any group. However, the ACT-D monodrug group had a higher incidence of leukopenia (grade 1 or 2) (59.38% vs 17.39%). The MTX regimen (5 days) might be the preferred treatment option. This study compared six monodrug chemotherapy regimens involving three common agents using network meta-analysis. We intended to settle the controversy regarding the chemotherapy regimen most suited for individual patients with low-risk GTN. We found that VP-16 (5 days) regimen might be the preferred option in terms of efficacy when the complete response is used as the endpoint of the study, the probability ranking is about 99%, which is significantly higher than the other five chemotherapy options.

Network meta-analysis was performed to compare the efficacy outcome of six monodrug chemotherapy regimens in GTN, with a complete response rate as the endpoint. There may be other single-drug regimens or different ways of using the same drug regimen, which were not included in the analysis. For example, the MITO study compared clinical outcomes of patients diagnosed with low-risk gestational trophoblastic neoplasia (GTN) receiving intramuscular methotrexate 50 mg total dose/day versus 1 mg/kg/day in a 8-day methotrexate/folinic acid (MTX/FA) regimen. Because the both regimens in this study are MTX (8 days) regimen, the study was excluded (34). At the same time, we should also consider the feasibility of the treatment plan, applicability and disturbance to patients. Need to investigate patients’ personal feelings. Choose a treatment plan based on multiple factors.

This study had certain limitations despite the clinical guidance it might provide. First, the included studies were mostly retrospective and of moderate quality. Considerable heterogeneity was found in the sample size across the included studies, which were published over a long period, leading to the risk of bias. Besides, we only included studies written in English at the expense of the loss of studies written in other languages. The conclusions drawn from the limited number of studies might contain some biases. The only endpoint discussed in the present study was the complete response rate. We did not analyze the incidence and severity of adverse reactions across the chemotherapeutic agents and dosing regimens, which might have affected the accuracy of the conclusions. The first-line monodrug chemotherapy regimens for low-risk GTN might differ in the incidence and severity of side effects. However, nearly all patients tolerated the associated adverse reactions. Very few cases of intolerance to adverse reactions were reported. The aforementioned results indicated that a complete response rate might be a more useful efficacy outcome compared with the incidence and severity of adverse reactions. These findings need to be further confirmed using high-quality evidence.

To conclude, we performed a network meta-analysis to compare the efficacy of six monodrug chemotherapy regimens in low-risk GTN. The evidence suggested that the VP-16 (5 days) regimen might be the preferred option in terms of efficacy, followed by ACT-D (5 days), MTX (5 days), ACT-D (1.25 mg/m^2^), MTX (8 days), and MTX (30–50 mg/m^2^). However, our conclusions should be verified through high-quality RCTs involving a large sample size. There are currently multiple registered clinical studies in progress, and the results of these studies will give us more clinical guidance (35).

## Author contributions

FZ: Conceptualization, Investigation, Methodology, Project administration, Resources, Supervision, Validation, Writing – review & editing. LK: Conceptualization, Data curation, Formal analysis, Methodology, Project administration, Resources, Software, Writing – original draft.

## Supplementary material

Supplementary Figure 1 GTN-node splitting analysis of inconsistency (**A**: in all study, **B**: in RCT). **(A)** ACT-D (10 ug/kg per day intravenously for 5 days,every 2 weeks), **(B)** MTX(1 mg/kg per day on days 1, 3, 5, and 7, alternating with intramuscular folinic acid 0.1 mg/kg per day on days 2, 4, 6, and 8, every two weeks), **(C)** pulse Act-D(pulse actinomycin-D (1.25 mg/m2) once every 14 days with a maximum dose of 2 mg), **(D)** MTX(0.4mg/kg 5 day), **(E)** VP-16(2.0mg/kg 5 day), **(F)** MTX(30 mg/m2/weekly).

Supplementary Figure 2 analysis of heterogeneity(**A**: in all study, **B**: in RCT). **(A)** ACT-D (10 ug/kg per day intravenously for 5 days,every 2 weeks), **(B)** MTX(1 mg/kg per day on days 1, 3, 5, and 7, alternating with intramuscular folinic acid 0.1 mg/kg per day on days 2, 4, 6, and 8, every two weeks), **(C)** pulse Act-D(pulse actinomycin-D (1.25 mg/m2) once every 14 days with a maximum dose of 2 mg), **(D)** MTX(0.4mg/kg 5 day), **(E)** VP-16(2.0mg/kg 5 day), **(F)** MTX(30 mg/m2/weekly).
