# Supplementary figures and images for: First-line monodrug chemotherapy in low-risk gestational trophoblastic neoplasia: a network meta-analysis
Source: Front Oncol. 2024 Jan 5;13:1276771. doi: 10.3389/fonc.2023.1276771 (PMC10796812; doi:10.3389/fonc.2023.1276771)

## Slide 1
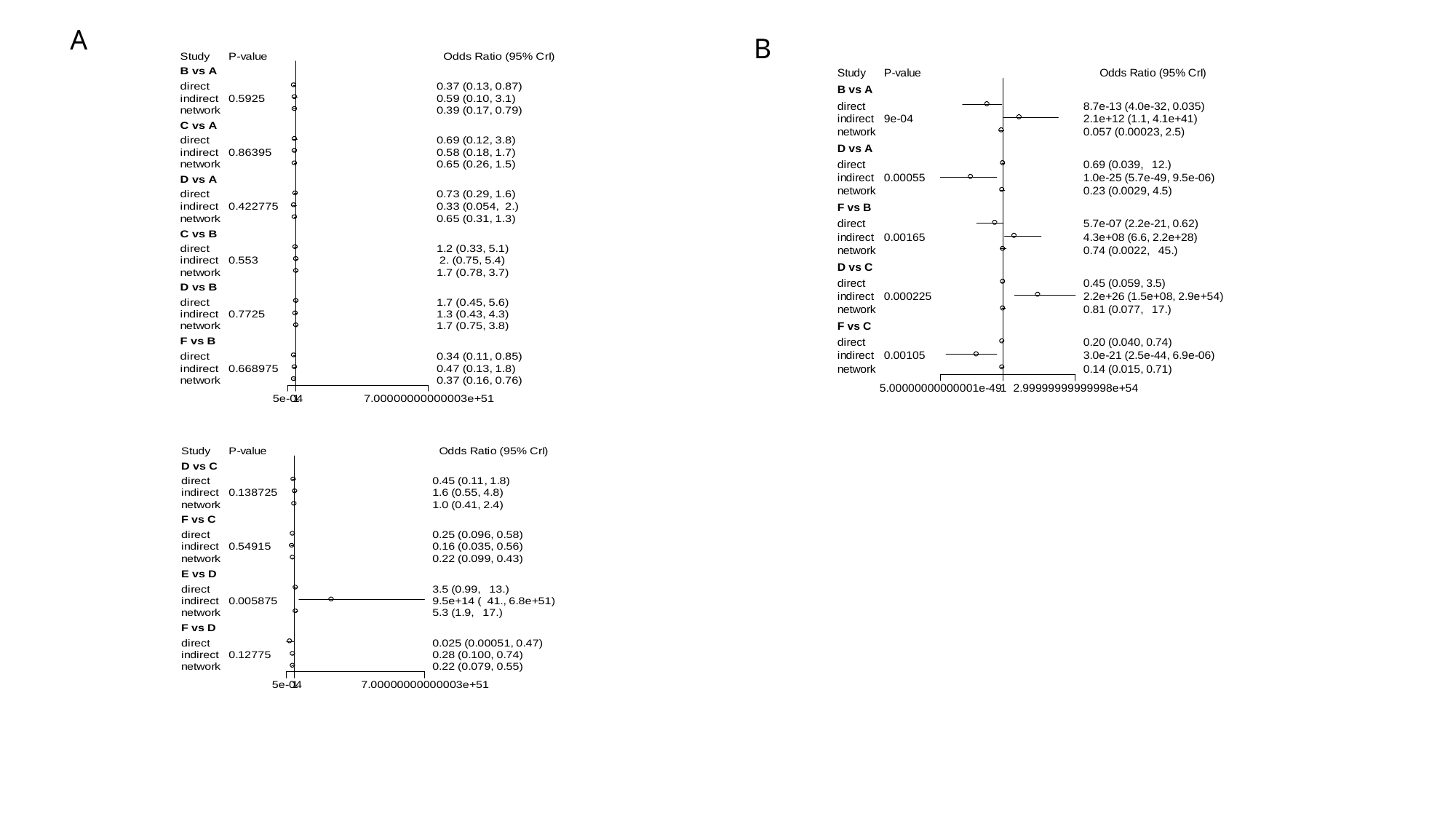

A
B

Supplement: Supplementary Figure 1 — GTN-node splitting analysis of inconsistency (A: in all study, B: in RCT). (A) ACT-D (10 ug/kg per day intravenously for 5 days,every 2 weeks), (B) MTX(1 mg/kg per day on days 1, 3, 5, and 7, alternating with intramuscular folinic acid 0.1 mg/kg per day on days 2, 4, 6, and 8, every two weeks), (C) pulse Act-D(pulse actinomycin-D (1.25 mg/m2) once every 14 days with a maximum dose of 2 mg), (D) MTX(0.4mg/kg 5 day), (E) VP-16(2.0mg/kg 5 day), (F) MTX(30 mg/m2/weekly). [file Presentation_1.pptx]

## Slide 1
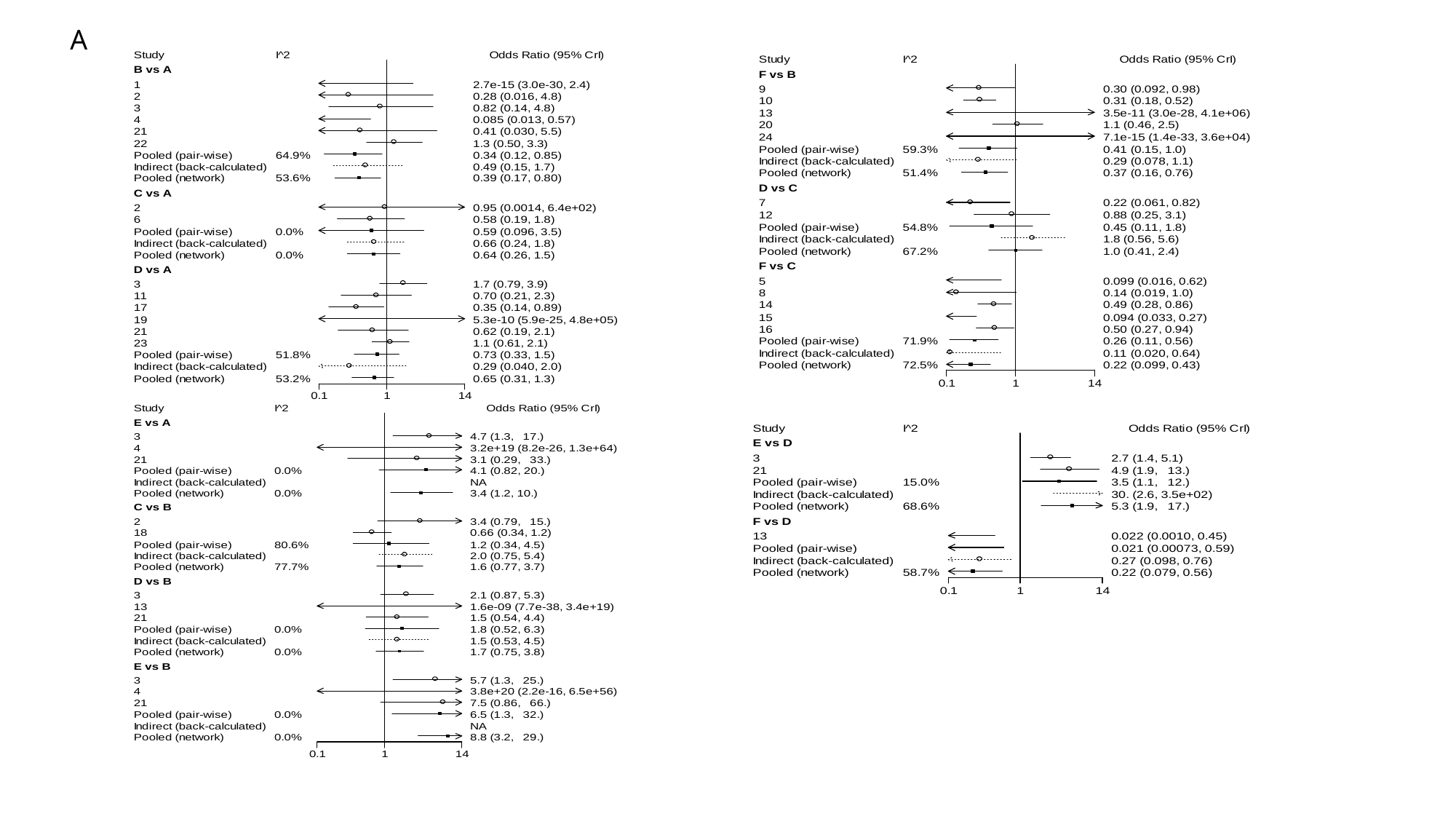

A

## Slide 2
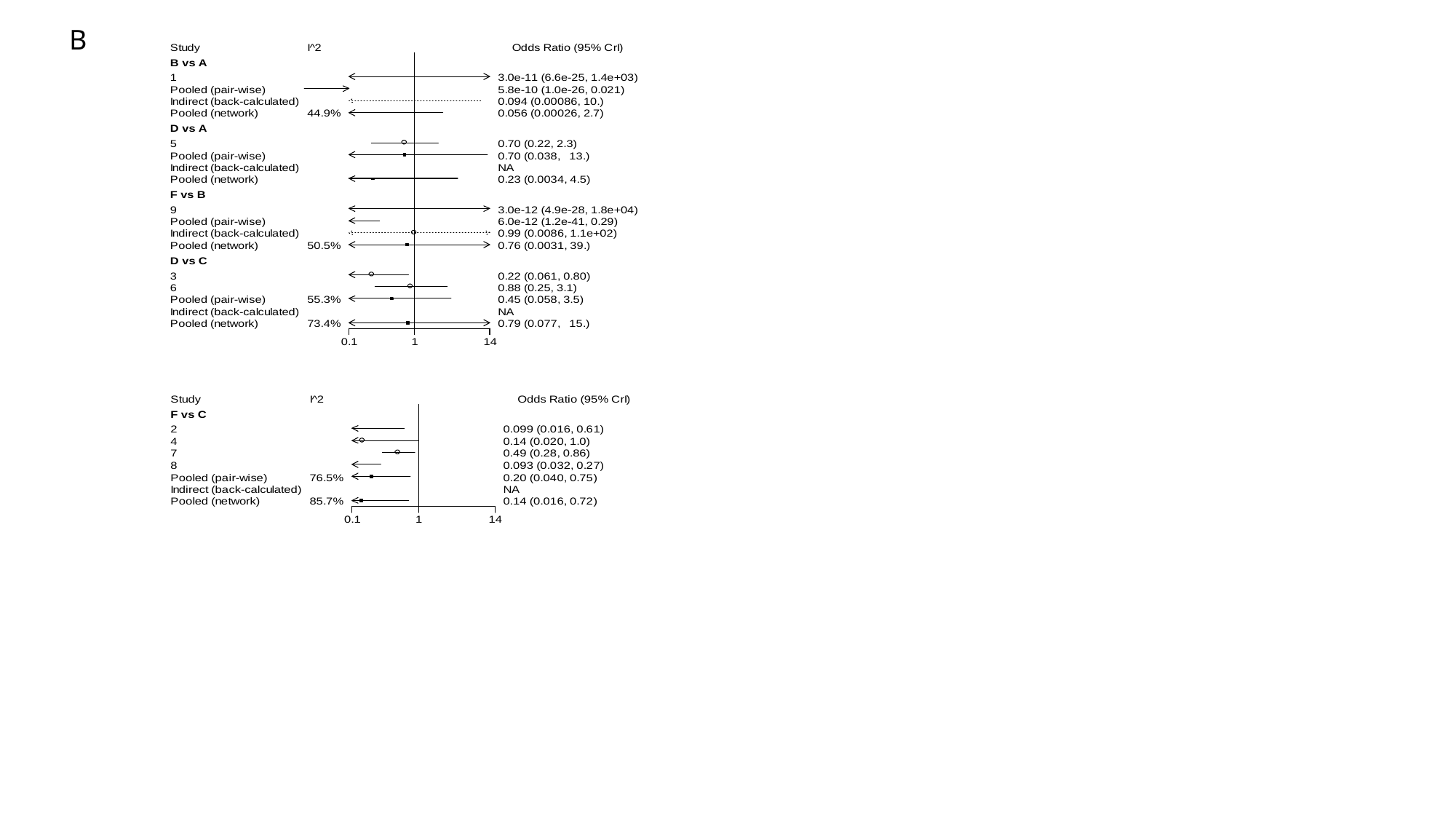

B

Supplement: Supplementary Figure 2 — analysis of heterogeneity(A: in all study, B: in RCT). (A) ACT-D (10 ug/kg per day intravenously for 5 days,every 2 weeks), (B) MTX(1 mg/kg per day on days 1, 3, 5, and 7, alternating with intramuscular folinic acid 0.1 mg/kg per day on days 2, 4, 6, and 8, every two weeks), (C) pulse Act-D(pulse actinomycin-D (1.25 mg/m2) once every 14 days with a maximum dose of 2 mg), (D) MTX(0.4mg/kg 5 day), (E) VP-16(2.0mg/kg 5 day), (F) MTX(30 mg/m2/weekly). [file Presentation_2.pptx]
