# Supplementary material for: First-line monodrug chemotherapy in low-risk gestational trophoblastic neoplasia: a network meta-analysis
Source: Front Oncol. 2024 Jan 5;13:1276771. doi: 10.3389/fonc.2023.1276771 (PMC10796812; doi:10.3389/fonc.2023.1276771)
Supplement: Supplementary file 3 [file Table_1.docx]

table s 1. inconsistency model

| ACT-D(1.25mg/m2) | 1.53(0.54,4.68) | 0.23(0.10,0.48) | 0.79(0.22,2.34) | 0.67(0.25,1.92) | 5.50(1.33,26.28) |
| --- | --- | --- | --- | --- | --- |
| 0.65(0.21,1.84) | ACT-D(5days) | 0.14(0.03,0.44) | 0.68(0.30,1.41) | 0.37(0.08,1.25) | 3.43(0.64,15.26) |
| 4.28(2.07,10.04) | 7.26(2.27,29.29) | MTX(30-50mg/m2) | 4.88(1.56,19.00) | 2.81(1.22,7.29) | 26.34(7.47,121.57) |
| 1.26(0.43,4.52) | 1.47(0.71,3.33) | 0.21(0.05,0.64) | MTX(5days) | 0.67(0.16,5.18) | 5.45(1.57,21.77) |
| 1.49(0.52,4.06) | 2.68(0.80,12.83) | 0.36(0.14,0.82) | 1.49(0.19,6.29) | MTX(8days) | 9.36(3.34,31.33) |
| 0.18(0.04,0.75) | 0.29(0.07,1.56) | 0.04(0.01,0.13) | 0.18(0.05,0.64) | 0.11(0.03,0.30) | VP-16(5days) |

table s 2 results- node-splitting analysis of inconsistency

| name | direct effect | indirect effect | overall | p value |
| --- | --- | --- | --- | --- |
| MTX(5days),VP-16(5days) | 1.26(0.08,2.50) | 29.54(4.89,119.58) | 1.66(0.64,2.80) | 0 |
| MTX(5days),MTX(8days) | -0.39(-1.55,0.92) | -0.80(-1.93,0.17) | -0.51(-1.31,0.26) | 0.6 |
| MTX(30-50mg/m2),MTX(8days) | 1.06(0.11,2.12) | 0.96(-0.28,2.27) | 0.99(0.28,1.82) | 0.9 |
| MTX(30-50mg/m2),MTX(5days) | 3.07(0.22,6.94) | 1.40(0.42,2.48) | 1.51(0.59,2.53) | 0.28 |
| ACT-D(5days),MTX(8days) | -1.05(-1.99,-0.24) | -0.21(-1.71,1.12) | -0.95(-1.76,-0.24) | 0.28 |
| ACT-D(5days),MTX(5days) | -0.30(-1.13,0.43) | -1.01(-2.54,0.52) | -0.43(-1.18,0.22) | 0.4 |
| ACT-D(1.25mg/m2),MTX(8days) | -0.17(-1.53,1.09) | -0.69(-1.65,0.21) | -0.50(-1.30,0.23) | 0.51 |
| ACT-D(1.25mg/m2),MTX(5days) | -0.82(-2.21,0.53) | 0.50(-0.56,1.57) | 0.01(-0.85,0.88) | 0.13 |
| ACT-D(1.25mg/m2),MTX(30) | -1.39(-2.34,-0.53) | -1.84(-3.36,-0.05) | -1.50(-2.29,-0.83) | 0.57 |
| ACT-D(5days),ACT-D(5days) | 0.45(-1.19,2.12) | 0.43(-0.58,1.51) | 0.44(0.41,1.35) | 0.99 |
